# Anti-Inflammatory and Antinociceptive Activities of Untreated, Germinated, and Fermented Mung Bean Aqueous Extract

**DOI:** 10.1155/2014/350507

**Published:** 2014-06-19

**Authors:** Norlaily Mohd Ali, Hamidah Mohd Yusof, Swee-Keong Yeap, Wan-Yong Ho, Boon-Kee Beh, Kamariah Long, Soo-Peng Koh, Mohd Puad Abdullah, Noorjahan Banu Alitheen

**Affiliations:** ^1^Department of Cell and Molecular Biology, Faculty of Biotechnology and Biomolecular Sciences, Universiti Putra Malaysia, 43400 Serdang, Selangor, Malaysia; ^2^Institute of Bioscience, Universiti Putra Malaysia, 43400 Serdang, Selangor, Malaysia; ^3^School of Biomedical Sciences, The University of Nottingham Malaysia Campus, Jalan Broga, 43500 Semenyih, Selangor, Malaysia; ^4^Department of Bioprocess Technology, Faculty of Biotechnology and Biomolecular Sciences, Universiti Putra Malaysia, 43400 Serdang, Selangor, Malaysia; ^5^Biotechnology Research Centre, Malaysian Agricultural Research and Development Institute (MARDI), 43400 Serdang, Selangor, Malaysia

## Abstract

Evaluation of anti-inflammatory and antinociceptive activities of untreated mung bean (MB), germinated mung bean (GMB), and fermented mung bean (FMB) was performed on both *in vitro* (inhibition of inflammatory mediator, nitric oxide(NO)) and *in vivo* (inhibition of ear oedema and reduction of response to pain stimulus) studies. Results showed that both GMB and FMB aqueous extract exhibited potent anti-inflammatory and antinociceptive activities in a dose-dependent manner. *In vitro* results showed that GMB and FMB were potent inflammatory mediator (NO) inhibitors at both 2.5 and 5 mg/mL. Further *in vivo* studies showed that GMB and FMB aqueous extract at 1000 mg/kg can significantly reduce ear oedema in mice caused by arachidonic acid. Besides, both 200 mg/kg and 1000 mg/kg concentrations of GMB and FMB were found to exhibit potent antinociceptive effects towards hotplate induced pain. With these, it can be concluded that GMB and FMB aqueous extract exhibited potential anti-inflammatory and antinociceptive effects.

## 1. Introduction

Inflammation is a host's defence mechanism against infection, foreign stimulus, and tissue damage by activating the cellular immune responses that increase stimulation of proinflammatory mediators such as nitric oxide (NO) and various cytokines. However, excessive production of inflammatory mediators will lead to chronic diseases such as rheumatoid arthritis and autoimmune diseases with hallmarks such as redness, swelling, pain, and loss of function [1]. On the other hand, nociceptive is a sensitization of pain transmitted by the nerves centrally or peripherally. Both processes are interconnected where in the event of inflammatory response, the mediators will also stimulate pain sensory [2].

Multiple drugs have been developed for the treatment of inflammation and nociceptive symptoms. Two typical examples are (a) nonsteroidal anti-inflammatory drug such as aspirin and dexamethasone and (b) prescribed drugs such as opioids and morphine. However, these types of drugs are commonly associated with undesired adverse effects. Due to ease of availability and less side effects, plant-derived compounds are of common interest in the search for alternative substitutes [3]. To the best of our knowledge, only single study has been conducted on the anti-inflammatory and antinociceptive properties of mung bean even though mung bean has been traditionally consumed as nutritional foods. Zhu et al. [4] have reported mung bean coat, which is rich in flavonoids that have positive anti-inflammatory effect against systemic inflammatory response.

Although many plant-derived products had studies conducted on their anti-inflammatory and antinociceptive properties, however, limited study was done to evaluate and compare the effects from mung bean (MB) whole seed, germinated mung bean (GMB), and fermented mung bean (FMB) aqueous extract. Hence, the objectives of this study were to evaluate and compare the* in vitro *and* in vivo *anti-inflammatory and antinociceptive properties of MB, GMB, and FMB aqueous extracts.

## 2. Materials and Methods

### 2.1. Chemicals and Reagents

Dulbecco's modified eagle medium (DMEM), L-glutamine, lipopolysaccharide (LPS), 3-(4,5-dimethylthiazol-2-yl)-2,5-diphenyl tetrazolium bromide (MTT), arachidonic acid, acetic acid, dexamethasone, and phosphate buffer saline were purchased from (Sigma, USA); penicillin/streptomycin, TypLE (GIBCO, USA), fetal bovine serum, Griess reaction (Invitrogen, USA), Interferon-gamma (IFN-*γ*) (Biolegend, USA), acetyl salicylic acid (ASA) (local pharmacy), 96-microtitre plate (TPP, Switzerland), serological pipettes, T-25 flasks, and 15 mL tubes (Corning, USA) were used. Murine macrophage cell line, RAW264.7, was obtained from American Type Culture Collection (ATCC, USA).

### 2.2. Preparation of MB, GMB, and FMB Aqueous Extracts

MB, GMB, and FMB aqueous extracts were prepared according to our previous study, Ali et al. [5]. In brief, FMB extract was prepared via solid-state fermentation using* Rhizopus *sp.strain 5351 (MARDI); meanwhile, GMB extract was prepared viaanaerobic germination. MB sample was directly ground into powder without prior fermentation or germination. Final extracts were obtained through water extraction and freeze-drying processes.

### 2.3. Cell Line and Culture Conditions

Murine cell line, RAW264.7, was maintained in DMEM culture media supplemented with 1% L-glutamine, 10% fetal bovine serum, and 1% penicillin/streptomycin in 25 cm^2^ flask at 37°C, 5% CO_2_ environment. Cells at 80% confluent, with viability >90% was harvested using TypLE (GIBCO, USA) for analysis.

### 2.4. MTT Cell Viability Screening

Cell viability was determined using MTT assay according to Fotakis and Timbrell [6] with slight modifications. Cells at a density of 2 × 10^5^ cell/mL were seeded to attach overnight and treated with various concentrations of MB, GMB, and FMB aqueous extracts (1.25, 2.5, 5, and 10 mg/mL) for 24, 48, and 72 hours. Then, MTT (5 mg/mL) solution was added to the culture and the tetrazolium salt was solubilized using DMSO. The absorbance was taken at 570 nm and percentage of total cells was calculated from the formula:

(1) %  of  cell  proliferation  =OD  sample−OD  controlOD  control×100.

### 2.5. Determination of Nitric Oxide (NO)

NO produced in RAW264.7 cells supernatant treated with extracts was determined using calorimetric Griess reaction. Same amount of cells density and treatment (2.5, 5 mg/mL) were performed as in previous MTT assay. To stimulate inducible NO synthase, LPS (10 *μ*g/mL) and IFN-*γ* (20 U/mL) were added to the culture media. Both LPS and IFN-*γ* were used to induce the inflammatory mediators (NO) and enzyme involved in inflammation such as COX-2 [7]. After 72 hours, 100 *μ*L of cells supernatant was transferred into a new 96-microtitre plate followed by addition of 100 *μ*L Griess reagent (1% sulphanilamide and 0.1% N-1-naphthylethylenediamine dihydrochloride in 2.5% polyphosphoric acid). The absorbance was taken at 540 nm using ELISA Reader (Bio-tek Instrument, USA). The data was normalized to standard using sodium nitrite and expressed as nitrite concentration (*μ*M) (Huang et al., 2012).

### 2.6. Animals

Male Balb/c mice aged 8–10 weeks old with average weight of 20–25 g were maintained under standard condition of temperature (22 ± 5°C) and humidity in animal house with 12 hours of light/dark cycle. Animals were allowed food and water* ad libitum*. Experiments were strictly conducted and approved by Animal Care and Use Committee, Universiti Putra Malaysia, (Ref: UPM/FPV/PS/3.2.1.551/AUP-R2).

#### 2.6.1. Arachidonic Acid-Induced Ear Oedema in Mice

Arachidonic acid-induced ear oedema in mice was investigated according to Lin et al. [7] with modifications. Mice were randomly divided into 8 groups (*n* = 6).

Both ears of the mice were topically applied with 100 *μ*L solution according to the following treatment groups:

 Group 1: normal saline
 Group 2: dexamethasone (30 mg/kg)
 Group 3: MB (200 mg/kg)
 Group 4: MB (1000 mg/kg)
 Group 5: GMB (200 mg/kg)
 Group 6: GMB (1000 mg/kg)
 Group 7: FMB (200 mg/kg)
 Group 8: FMB (1000 mg/kg).

After 1 hour of treatment, 100 *μ*L of arachidonic acid (0.5 mg/kg) dissolve in acetone was topically administered onto mice right ear and we waited for another hour. Mice were sacrificed by cervical dislocation and both of the ears were punched or sectioned 6 mm and weighed. The oedema was calculated by the difference between the weights of induced ear oedema (right ear) and the noninduced (left ear). The anti-inflammatory activity was expressed as the percentage of inhibition (%) exerted by the extracts in comparison to the control (saline) treated group.

#### 2.6.2. Heat-Induced Paw Licking in Mice

Heat induced pain was investigated according to Okokon et al. [8] with alterations. The hot plate with glass wall was used to introduce the heat to the mice and the response latencies were measured based on the time taken for the mice to start licking or shaking the hind paw or jumping. Mice fasted for 24 hours prior to experiment and randomly divided into 8 groups (*n* = 6). All mice were pretreated orally (p.o.) with 100 *μ*L solution 30 minutes prior to placement on hot plate according to the following grouping:

 Group 1: normal saline
 Group 2: ASA (400 mg/kg)
 Group 3: MB (200 mg/kg)
 Group 4: MB (1000 mg/kg)
 Group 5: GMB (200 mg/kg)
 Group 6: GMB (1000 mg/kg)
 Group 7: FMB (200 mg/kg)
 Group 8: FMB (1000 mg/kg).

Hot plate was maintained at (55 ± 2)°C and each animal was placed onto the heated surface one at a time. The time taken for each mouse to display response latencies was recorded as the index of response latency at time interval of 1 hour up to 6 hours. A 40 seconds cut-off time was strictly executed to prevent tissue damage. Time of response latency prior to administration of extracts was also recorded. Data was expressed as the reduction number of response latency between control and extracts-treated mice.

### 2.7. Statistical Analysis

All quantitative measurements were conveyed as mean ± standard deviation. Analyses were performed using one-way analysis of variance (ANOVA) and the group means were compared by Duncan's test. *P* values <0.05 were considered as statistically significant.

## 3. Results 

### 3.1. Cell Viability and Cytotoxicity

**Figure 1:**
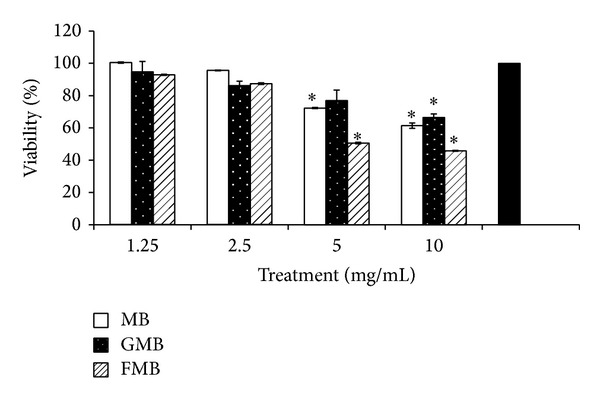
Cell viability of murine cells, RAW264.7, was determined at various concentrations of aqueous extract, MB, GMB, and FMB at 72 h of incubation. Note: Values are mean ± SEM of at least 3 replicates and significantly different from untreated (100% viability) (∗*P* < 0.05) by ANOVA and followed by Duncan's multiple range test.

The effect of MB, GMB, and FMB aqueous extracts on RAW264.7 cells viability was accessed using MTT assay at different incubation times. As depicted in Figure 1, following 72 hours of treatment, all extracts at 10 mg/mL exerted significant cytotoxicity effect against murine macrophage cells, RAW 264.7. FMB shows the highest inhibitory effect on RAW264.7 with 54% inhibition followed by MB (39%) and GMB (34%), respectively. Meanwhile, at lower range of dose (1.25 and 2.5 mg/mL), no significant inhibition was detected in all treated cells, which indicates that the extracts are safe to be applied on macrophage cells without causing extensive cell death. Also, the concentration near IC_50_ (2.3 mg/mL) value against cancer cells MCF-7 (unpublished data) showing minimal cytotoxic effect suggesting that the extracts are selective against normal cells RAW264.7. On the other hand, no significant inhibition of RAW264.7 cells was detected after treatment with extracts at 24 and 48 hours of incubation periods (data not shown).

### 3.2. NO Determination

**Figure 2:**
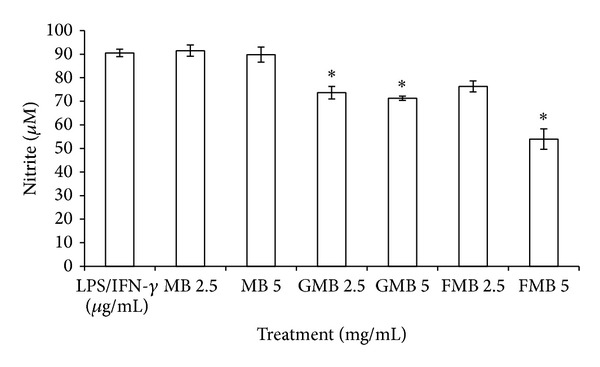
Effects of aqueous extracts MB, GMB, and FMB on nitrite concentration in LPS/IFN-*γ* stimulated RAW264.7 cells after 72 h of incubation. Note: Values are mean ± SEM of at least 3 replicates and significantly different from untreated (LPS/IFN-*γ*) (∗*P* < 0.05) by ANOVA and followed by Duncan's multiple range test.

The effect of MB, GMB, and FMB aqueous extracts on NO inhibition was accessed through the quantification of nitrite (NO_2_^−^) level in the cells supernatant. NO is one of the final product of NO oxidation. The murine macrophage RAW264.7 cells were induced with LPS/IFN-*γ* to significantly boost the production of NO. As shown in Figure 2, only GMB and FMB exerted inhibitory effects against NO at both concentrations. The inhibition of NO level after GMB treatment at 2.5 and 5 mg/mL was 18.6% and 21.6% inhibition, respectively. Similarly, FMB at 2.5 and 5 mg/mL was able to inhibit NO by 15.7 and 40.3%, respectively. There is possibility that high inhibition of NO by FMB (5 mg/mL) was partly contributed by cytotoxicity effects as seen in Figure 1. On the other hand, both concentrations of MB extract did not exert any inhibitory effect toward NO.

### 3.3. Inhibition of Arachidonic Acid-Induced Ear Oedema in Mice

**Figure 3:**
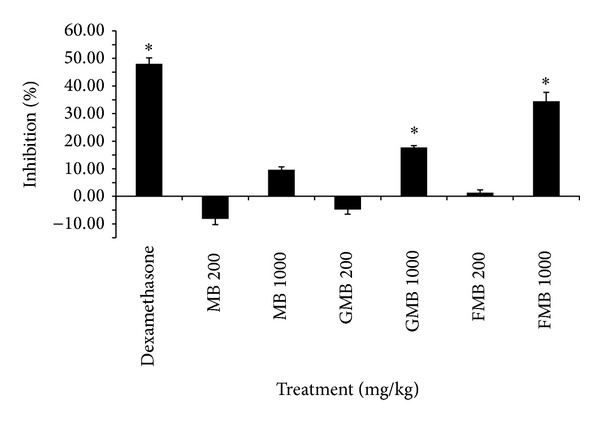
Inhibition effects of MB, GMB, and FMB aqueous extracts on mice ear oedema induced by arachidonic acid. Dexamethasone (30 mg/kg) was used as reference standard. Note: Values are mean ± SEM (*n* = 6) and significantly different from untreated (0% of inhibition) (∗*P* < 0.05) by ANOVA and followed by Duncan's multiple range test.

Arachidonic acid-induced ear oedema was performed to evaluate the* in vivo *anti-inflammatory activity of MB, GMB, and FMB aqueous extract. In this acute inflammation model, the percent of inhibition was accessed through the difference of oedema weight between left and right earlobes after external application of extracts. Arachidonic acid was topically applied to induce irritation and swelling, which led to oedema. As summarized in Figure 3, dexamethasone as reference drug exhibited the highest oedema inhibition effect followed by FMB (1000 mg/kg) and GMB (1000 mg/kg). MB (1000 mg/kg) and FMB (200 mg/kg) have minimal inhibitory effect whereas MB (200 mg/kg) and GMB (200 mg/kg) have no inhibitory effect observed. The results above complement the* in vitro* data suggesting that FMB and GMB extracts at high dose have anti-inflammatory effect on acute inflammation.

### 3.4. Hot Plate Assay

Hot plate assay was carried out to evaluate the antinociceptive effect of MB, GMB, and FMB aqueous extracts on heat-induced pain. The increase of latency period was assessed in evaluating the antinociceptive activity of extracts. Oral pretreatment with extracts led to significant increase of reaction time in hot plate test. As shown in Table 1, all extracts prolonged the response time of mice relative to heat stimulation with different response periods. After 90 minutes of extracts treatment, all mice showing remarkable endurance against heat induced pain. The response was noticed to be highest at high dose (1000 mg/kg) of FMB with response time 18 seconds during 90 minutes interval. Furthermore, GMB at low and high dose resulted in 18.2 and 18.4 seconds response time, respectively, at 120 minutes after intake of extract. Both FMB and GMB extracts are comparable with the reference drug, ASA. These results suggest that all extracts at certain interval time have antinociceptive effect against pain stimulus and have the potential as analgesia.

## 4. Discussion

Due to many inflammatory diseases such as rheumatoid arthritis, autoimmune disease, asthma, cancer, and other inflammatory-related diseases and their serious side effect, there has been increased interest in search of the novel and safe anti-inflammatory and antinociceptive agents [9]. In this study, we examined the* in vitro *and* in vivo *anti-inflammatory as well as antinociceptive activities of MB, GMB, and FMB aqueous extracts. In* in vitro* study, the viability of murine macrophage cells, RAW264.7, after treatment with extracts at 1.25, 2.5, 5, and 10 mg/mL was evaluated. Cytotoxicity effect of all extracts on cells was noticed only after prolonged incubation at high dose (10 mg/mL). The analysis was followed by NO inhibition study in which GMB and FMB extracts were able to inhibit the inflammatory mediator, NO by 15.7% and 40.3%, respectively.

The detection of inflammatory mediators is crucial in evaluating the anti-inflammatory effect of different extracts. One of the inflammatory mediators is NO released by an inducible form of nitric oxide synthase (iNOS) in activated macrophages [10]. The upregulation of NO stimulated by LPS and IFN-*γ* was used as a baseline to evaluate the ability of different extracts to inhibit the production of inflammatory mediator. Inhibition of NO secreted by activated macrophages during inflammatory response can be used to treat inflammation-related diseases [7]. Inflammation is a protective response against foreign stimulus that consists of the innate system of cellular, humoral responses followed by insult. Uncontrolled regulation of inflammatory responses can lead to various inflammatory diseases and pain (Gil et al., 2012). The results obtained were in complement with our previous findings where FMB and GMB showed an average of better hepatoprotective effects than MB at both 200 mg/kg and 1000 mg/kg in ethanol induced liver damage* in vivo* [5]. In liver damage, accumulation of NO was observed in response to ethanol induction. Thus, ability of FMB and GMB to attenuate the NO level in* in vivo *liver injury study should be parallel with the* in vitro *LPS/IFN-*γ*-stimulated macrophages.

We have previously reported that FMB and GMB contained higher amount of GABA and total amino acids compared to MB that may contribute to the hepatoprotective and antioxidant effects against alcohol liver injury [5]. Similarly, the increase of both bioactive compounds may also contribute and justify the NO inhibition effects in murine macrophages cells and inhibition of ear oedema in mice. This also could be due to the presence of active compounds such as gallic acid, vitexin, and isovitexin in both GMB and FMB products which could provide more potent antioxidant activities [11–13]. In addition, Montana et al. (2012) have claimed that antioxidant activity of plant flavonoids and phenolic acids is able to scavenge the reactive oxygen species (ROS), which mainly are involved in inflammation pathology.

The study was further continued with* in vivo *anti-inflammatory and antinociceptive analysis. In evaluating* in vivo *anti-inflammatory, acute inflammatory model in mice was obtained and the assessment was evaluated through the reduction of oedema weight after treatment. Both FMB and GMB extracts at 1000 mg/kg exhibited potent anti-inflammatory effect on acute inflammation diseases. Possible justification of better anti-inflammatory effects from both GMB and FMB at high does could be due to the presence of flavonoids and phenolic compounds that are highly expressed during germination and fermentation [14, 15].

Excessive inflammation can lead to nociceptive event in which stimulation of pain and tissue damage start to occur [1]. The antinociceptive study was evaluated using hot plate assay heat-induced pain. Results showed that all extracts especially GMB and FMB at high concentration were comparatively effective as the positive analgesic drug. ASA is a well-known nonsteroidal anti-inflammatory drug (NSAID) that is widely used for its anti-inflammatory and antinociceptive properties [16]. GABA is a type of neuron inhibitors in mammals, where studies have illustrated that it can act as anti-inflammatory agent [17]. The antinociceptive property from GMB and FMB could be due to the presence of *γ*-aminobutyric acid (GABA), which was found to increase in concentration during germination and fermentation processes [18].

The results show consistency between* in vitro *and* in vivo* anti-inflammatory studies. The results also agreed with those previously reported on anti-inflammatory activity of mung bean plant. A major active compound that is responsible for exhibiting the effect might be attributed to the flavonoids as described by Zhu et al. [4]. They have reported the anti-inflammatory properties of an aqueous extract of the mung bean coat against systemic inflammatory disease; sepsis and mass spectrometry analysis confirmed that the flavonoids responsible for the activity were vitexin and isovitexin [4]. In addition, Montana et al. have claimed that the antioxidant activity of plant flavonoids and phenolic acids are able to scavenge the reactive oxygen species (ROS), which mainly are involved in inflammatory pathology [9]. In conjunction to that, mung bean was reported to contain high amount of antioxidant component particularly flavonoids and phenolic compounds and even higher in germinated and fermented plant [15]; (Li et al., 2012).

## 5. Conclusion

In the evaluation of* in vitro *and* in vivo *anti-inflammatory and antinociceptive activities of MB, GMB, and FMB aqueous extracts, GMB and FMB aqueous extracts have exhibited potent anti-inflammatory activities and have potential as antinociceptive agents and analgesics. They also have the prospective to be commercially marketed as supplement or developed as drug for inflammatory diseases treatment.

## Figures and Tables

**Table 1:** Effect of oral administration of MB, GMB, and FMB aqueous extracts on heat induced paw licking in mice. Acetyl salicylic acid (ASA) (400 mg/kg) was used as reference drug. (Unit = seconds.)

Treatment	Dose (mg/kg)	Latency period (minute)				
		Before treatment	After treatment			
		0	30	60	90	120
Control (normal saline)	0	13.0 ± 0.10	11.2 ± 0.70	11.7 ± 0.91	10.8 ± 0.30	9.5 ± 0.71
MB	200	13.9 ± 0.75	13.8 ± 1.07	13.7 ± 0.95	14.3 ± 0.50∗	13.3 ± 0.80
MB	1000	13.8 ± 0.10	13.3 ± 0.90	14.5 ± 0.25∗	15.9 ± 0.80∗	12.6 ± 1.05
GMB	200	13.5 ± 0.38	14.9 ± 0.20∗	13.9 ± 0.51	15.4 ± 0.84∗	18.2 ± 0.70∗
GMB	1000	13.3 ± 0.26	13.8 ± 0.85	14.4 ± 0.75∗	17.0 ± 1.09∗	18.4 ± 0.68∗
FMB	200	13.7 ± 0.55	14.1 ± 0.15	15.8 ± 1.03∗	15.2 ± 1.00∗	12.5 ± 0.55
FMB	1000	13.5 ± 0.75	14.2 ± 1.30	15.5 ± 0.35∗	18.0 ± 1.29∗	14.1 ± 0.65
ASA	400	13.4 ± 0.60	14.5 ± 1.00∗	16.8 ± 0.53∗	17.2 ± 0.80∗	15.1 ± 1.70∗

Note: Values are mean ± SEM (*n* = 6) and significantly different from control (∗*P* < 0.05) by ANOVA and followed by Duncan's multiple range test.

## Abbreviations

MB: Mung bean
GMB: Germinated mung bean
FMB: Fermented mung bean
DMEM: Dulbecco's modified eagle medium
LPS: Lipopolysaccharide
MTT: 3-(4,5-Dimethylthiazol-2-yl)-2,5-diphenyl tetrazolium bromide
NO: Nitric oxide
ASA: Acetyl salicylic acid
IFN-*γ*: Interferon-gamma
GABA: *γ*-Aminobutyric acid.
